# Assessment of Statistical Process Control Based DVH Action Levels for Systematic Multi-Leaf Collimator Errors in Cervical Cancer RapidArc Plans

**DOI:** 10.3389/fonc.2022.862635

**Published:** 2022-05-18

**Authors:** Hanyin Zhang, Wenli Lu, Haixia Cui, Ying Li, Xin Yi

**Affiliations:** Department of Oncology, The First Affiliated Hospital of Chongqing Medical University, Chongqing, China

**Keywords:** cervical cancer RapidArc plan, 3D quality assurance, DVH-based action levels, statistical process control, systematic MLC errors

## Abstract

**Background:**

In the patient-specific quality assurance (QA), DVH is a critical clinically relevant parameter that is finally used to determine the safety and effectiveness of radiotherapy. However, a consensus on DVH-based action levels has not been reached yet. The aim of this study is to explore reasonable DVH-based action levels and optimal DVH metrics in detecting systematic MLC errors for cervical cancer RapidArc plans.

**Methods:**

In this study, a total of 148 cervical cancer RapidArc plans were selected and measured with COMPASS 3D dosimetry system. Firstly, the patient-specific QA results of 110 RapidArc plans were retrospectively reviewed. Then, DVH-based action limits (AL) and tolerance limits (TL) were obtained by statistical process control. Secondly, systematic MLC errors were introduced in 20 RapidArc plans, generating 380 modified plans. Then, the dose difference (%DE) in DVH metrics between modified plans and original plans was extracted from measurement results. After that, the linear regression model was used to investigate the detection limits of DVH-based action levels between %DE and systematic MLC errors. Finally, a total of 180 test plans (including 162 error-introduced plans and 18 original plans) were prepared for validation. The error detection rate of DVH-based action levels was compared in different DVH metrics of 180 test plans.

**Results:**

A linear correlation was found between systematic MLC errors and %DE in all DVH metrics. Based on linear regression model, the systematic MLC errors between -0.94 mm and 0.88 mm could be caught by the TL of PTV_95_ ([-1.54%, 1.51%]), and the systematic MLC errors between -1.00 mm and 0.80 mm could also be caught by the TL of PTV_mean_ ([-2.06%, 0.38%]). In the validation, for original plans, PTV_95_ showed the minimum error detection rate of 5.56%. For error-introduced plans with systematic MLC errors more than 1mm, PTV_mean_ showed the maximum error detection rate of 88.89%, and then was followed by PTV_95_ (86.67%). All the TL of DVH metrics showed a poor error detection rate in identifying error-induced plans with systematic MLC errors less than 1mm.

**Conclusion:**

In 3D quality assurance of cervical cancer RapidArc plans, process-based tolerance limits showed greater advantages in distinguishing plans introduced with systematic MLC errors more than 1mm, and reasonable DVH-based action levels can be acquired through statistical process control. During DVH-based verification, main focus should be on the DVH metrics of target volume. OARs in low-dose regions were found to have a relatively higher dose sensitivity to smaller systematic MLC errors, but may be accompanied with higher false error detection rate.

## Introduction

As a rapidly developed and increasingly used technique, volumetric modulated arc therapy (VMAT) is capable of delivering a high conformal dose distribution in a short delivery time (1, 2). As Varian’s commercial implementation of the VMAT algorithm proposed by Otto in 2008 (3), RapidArc incorporates concomitant continuous gantry rotation, dynamic movement of multileaf collimators (MLC) and variable dose rate (4). Due to rotational delivery feature and increased complexity in planning and delivery, RapidArc poses a great challenge to treatment planning system (TPS) and linac performance. In order to ensure the safety of patient, patient-specific quality assurance (QA) must be implemented before the delivery of RapidArc plan. Action level is often used to determine whether the delivered plan is appropriate for the treatment of a patient. If the result of a patient-specific QA exceeds a predetermined action level, the plan will be delayed until the source of error is identified or the treatment is re-planned (5).

Nowadays, gamma analysis is the most widely used method for patient-specific QA. However, the results of gamma analysis have not been found to correlate well with clinically relevant metrics (such as the estimated deviations in dose volume histograms) (6). Under this circumstance, some researchers have incorporated DVH information into patient-specific QA results. In 2011, Nemls et al. (7) had proposed that “false negative” and “false positive” in conventional QA results could be revealed by DVH metrics. However, they failed to clarify how to set up action levels of DVH metrics reasonably and scientifically. In their following research, they have suggested that the difference of DVH metrics exceeding 5% represents a clinical implication and further studies about DVH-based action levels are required (8). Subsequently, different DVH-based action levels ranging from 2% to 5% have been proposed in a significant number of publications (4, 5, 9–11). Among them, 3% or 5% has been the most commonly used DVH-based action levels. In AAPM TG-119 report (12), the action levels of 4.5% and 4.7% have been recommended for point dose measurements in target and low-dose regions, respectively. In AAPM TG-218 report (13), it has been emphasized that universal action levels do not fit every institution, every case or every structure. In addition, some researchers (9, 14) have attempted to adopt specific DVH-based action levels for different structures, such as 3% for target volume and 5% for OARs. Ruurd Visser et al. (15) have also set DVH-based action levels of 2%-4% to evaluate nine structure types in head and neck IMRT treatment plans. They have found that the proposed DVH-based action levels are too strict for some structures, such as target volume or OARs near the target volume. In our previous study (16), the most commonly used DVH-based action level (3% or 5%) was found to be unsuitable for all structures. Because QA results may be affected by QA equipment, delivery system, tumor sites, plan’s complexity and so on. Given the above, it is a challenge to set a reasonable and scientific DVH-based action level according to actual situation.

Actually, AAPM TG-218 report has also focused on the issue that general action levels do not fit all situations, and recommended that locally defined action levels could be achieved by setting process-based tolerance and action limits. The locally defined action levels should ultimately be specific to local equipment, processes and case types as well as the experience of local physicist (17). Process-based tolerance and action limits are derived from statistical process control (SPC). SPC is a powerful analytical decision-making tool for monitoring production processes, achieving stability, and reducing variability (18). It mainly consists of two steps (19). The first step is to collect empirical data to determine the control limits, and the second step is to monitor the process by using a control chart to detect whether the data exceed the control limits. As an important process to ensure the safety of patients and fidelity of treatment, SPC could also be used to monitor the results of patient-specific QA. Actually, the application of SPC for analyzing QA process in radiotherapy has been growing over recent years and achieved good results (18–23). However, SPC is primarily utilized for obtaining locally defined action levels about gamma passing rates. No studies to date have rigorously validated the process-based DVH action levels for RapidArc plans. In addition, a variety of structures or DVH metrics are included in radiotherapy plan, so the process-based DVH action levels can also be calculated in different structures or DVH metrics. Therefore, process-based tolerance and action limits should be promising and viable options for obtaining DVH-based action levels.

In current radiotherapy practice, DVH is a critical clinically relevant parameter that is finally used to determine the safety and effectiveness of radiotherapy, and it has indeed improved the correlation between patient-specific QA results and clinically relevant metrics, but a consensus on DVH-based action levels has not been reached yet. In this study, different magnitude of systematic MLC errors were introduced in cervical cancer RapidArc plans, and a comprehensive and systematic evaluation was performed between process-based DVH action levels and commonly used DVH-based action levels. By doing so, we aimed to explore reasonable DVH-based action levels and optimal DVH metrics in detecting systematic MLC errors.

## Materials and Methods

### Overview

**Figure 1:**
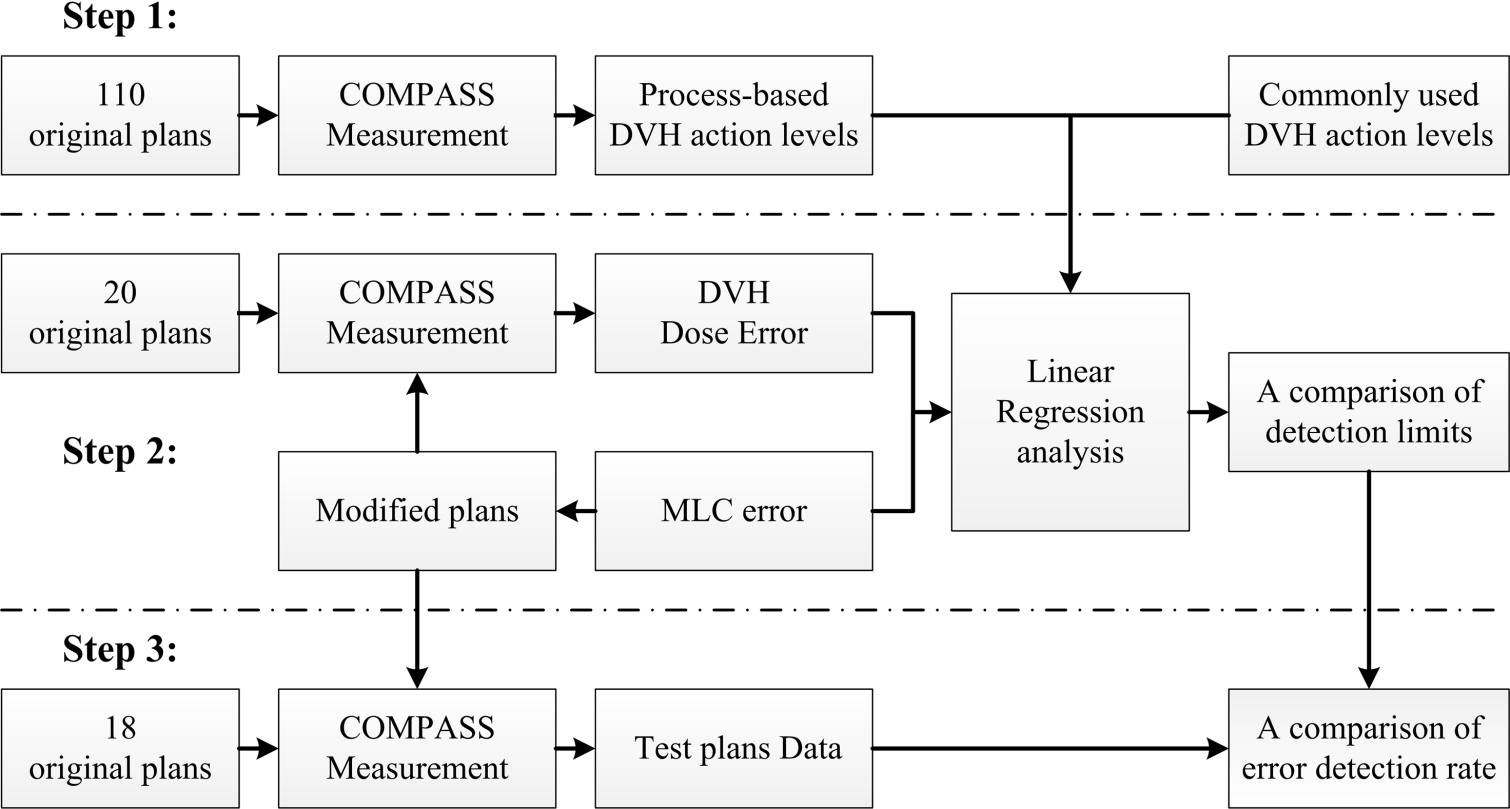
Flowchart of DVH-based action levels evaluation.

In this study, we designed a method to evaluate the detectability of different DVH-based action levels in systematic MLC errors. The method was summarized as below and schematized in **Figure 1**. It was mainly composed of three steps. Firstly, 110 RapidArc plans were selected and their patient-specific QA results were reviewed. DVH-based action levels were acquired by statistical process control. Meanwhile, other commonly used DVH-based action levels were also collected for comparison. Secondly, 20 other RapidArc plans were selected, in which different magnitude of systematic MLC errors were introduced. QA measurement was performed for all original and modified plans by COMPASS 3D dosimetry system. Then, the dose errors (%DE) of DVH information in specific structures were extracted from the measurement-based QA results of all original plans and modified plans. After that, the %DE of DVH metrics and the magnitude of systematic MLC errors were analyzed by linear regression analysis. The detection limits in DVH-based action levels mentioned in first step were investigated and compared by linear regression model to find out reasonable action levels for detecting systematic MLC errors. Thirdly, a total of 180 plans, including 162 error-introduced plans and 18 original plans, were prepared for validation. The error detection rate of DVH action levels was compared in different DVH metrics in order to explore the optimal DVH metrics to detect systematic MLC errors.

### Treatment Planning and Delivery

From 2017 to 2020, a total of 148 cervical cancer RapidArc plans were selected for this retrospective study. The aim of these enrolled plans was to deliver dose equal to 50Gy (in 25 fractions) to 95% of the planning target volume (PTV), while simultaneously meeting the plan acceptance for critical structures (bladder, rectum and femoral heads). All plans contained two full arcs: CCW with a start angle of 178° and collimator angle of 350°, and CW with a start angle of 182° and collimator angle of 10°. Each plan was calculated on a 2.5 mm isotropic dose grid with anisotropic analytical algorithm (AAA) through Eclipse v13.5 (Varain Medical Systems, Palo Alto, CA, USA). These plans were delivered by a 6 MV linear accelerator (Unique, Varian Medical Systems, Palo Alto, CA, USA) equipped with a millennium 120 multileaf collimators. All plans were delivered to COMPASS 3D dosimetry system for dose verification before patient treatment. In dose verification, the enrolled plans must meet the following criteria: 1. The gamma passing rates (GPRs) (3%/2mm, 10% dose threshold) should be greater than 95%. 2. The mean gamma index (GI) (3%/2mm, 50% dose threshold) should be smaller than 0.5.

### COMPASS 3D Dosimetry System

The COMPASS 3D dosimetry system (IBA Dosimetry, Germany) was used to generate independent data for 3D dose verification. This system is composed of a MatriXX 2D array and the analysis software COMPASS V4.1. The MatriXX 2D array consists of 1020 vented pixel parallel plate ionization chambers with a spatial resolution of 7.62mm (center-to-center distance of chambers). In dose verification, dose distribution in patient CT could be determined by either a dose calculation (model-based) or a dose reconstruction (measurement-based).

In this study, QA data were focused on COMPASS measurement-based QA results. In the measurement-based QA, 3D dose distribution was reconstructed based on fluence measured by MaritXX 2D array and dose calculation was performed by collapsed cone convolution (CCC) algorithm on patient’s CT. In this situation, data transfer and linac behavior were taken into account during comparing the TPS calculated dose with the COMPASS reconstructed dose. The QA data were acquired strictly according to operation manual. MatriXX was calibrated at each QA session to remove drift in linac output. Of course, routine QA procedure was rigorously performed prior to every COMPASS measurement. In addition, stability of linac delivery was verified at each QA session using a standard head and neck (H&N) RapidArc treatment plan.

### MLC Error Introduction

**Figure 2:**
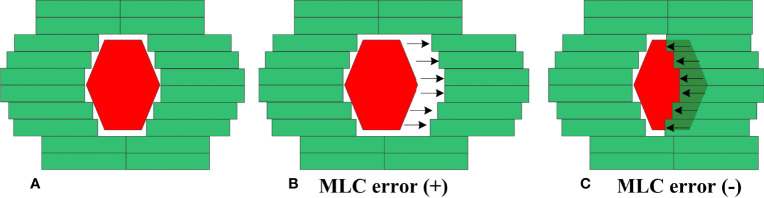
Creation of MLC error-induced RapidArc plan. **(A)** original plan **(B)** error-induced plan with positive MLC errors **(C)** error-induced plan with negative MLC errors.

MLC errors mainly include individual MLC positional errors, random MLC positional errors and systematic positional errors. Individual MLC positional errors or random MLC positional errors have little dosimetric effect on RapidArc plans (24). As such, systematic MLC positional errors were introduced in this study. An in-house Python program based on Pydicom (version 2.1.2) was applied to introduce systematic MLC errors by manipulation of DICOM RT files. In total, 20 plans were randomly selected from enrolled RapidArc plans. As described in **Figure 2**, the introduced systematic MLC errors included increase and decrease of the distance between leaf pairs in beam field. The magnitude of systematic MLC errors included ±0.2mm, ± 0.4mm, ± 0.6mm, ± 0.8mm, ± 1mm, ± 2mm, ± 3mm, ± 4mm, ± 5mm, respectively. If any magnitude of MLC errors has led to a negative leaf gap in some leaf pairs, the gap of relevant leaf pair would have been set to the minimal dynamic leaf gap of linac. After systematic MLC errors were introduced into the original plans, the modified RT files were re-imported into TPS for dose calculation. 19 plans were generated per patient (1 baseline plan plus 18 different magnitude of MLC error-introduced plans), so there were 380 plans in total. In the same manner, a total of 180 plans, including 162 error-introduced plans and 18 original plans, were also generated as a validation dataset.

### Data Analysis

With the measurement-QA results of COMPASS system, the %DE of DVH information between original plans and modified plans was calculated as follows:


(1) $$\%DE=\frac{D_{mod}-D_{ori}}{D_{ori}}\times 100\%$$

**%*DE*** refers to the relative dose percentage change between the original plan dose (*D*_ori_) and the measured dose from the modified plans (*D_mod_*). The extracted DVH metrics included the dose received by 95% of PTV (PTV_95_), the dose received by 5% of PTV (PTV_5_), the mean dose of PTV (PTV_mean_), the mean dose of bladder (Bladder_mean_), rectum (Rectum_mean_) and femoral heads (FH_mean_). These QA results were reviewed for statistical analysis. The Kolmogorov-Smirnov test was performed to determine whether a data set of each DVH metric was well-modeled by the normal distribution (*p*>0.05).

Once the %DE of DVH metrics was collected from the measurement-QA results of 110 RapidArc plans, process-based DVH action levels were obtained by statistical process control. This method was recommended in AAPM TG-218 report, and the general formula is as follows:


(2) $$\{AL=T\pm \beta \sqrt{\sigma^{2}+{\left(x-T\right)}^{2}}TL=x\pm 2.660\cdot \bar{mR}$$

***AL*** is the action limits. ***T*** refers to the process target value; ***σ^2^*** and *x* indicate the process variance and process mean respectively. As a constant, ***β*** is set to 6.0 in this equation. ***TL*** is the tolerance limits. Calculated by equation $\bar{mR}=\frac{1}{n-1}\sum_{i=2}^{n}\left|x_{i}-x_{i-1}\right|$ , $\bar{mR}$ indicate the moving range. *n* is the total number of measurements. The details were explicitly explained in AAPM TG-218 report (13).

A linear regression analysis was implemented between the %DE of DVH metrics and introduced MLC errors. By linear regression analysis, the slope, indicating the dose percentage change per mm of MLC error in each DVH metric, was checked. In the study, all presented DVH-based action levels were investigated by linear regression model. The detectability of DVH-based action levels was evaluated through the theoretical detection limit in catching system MLC errors. The optimal DVH-based action levels in different DVH metrics were also examined by 180 test RapidArc plans. The error detection rate of DVH-based action levels was compared in different DVH metrics. The original plans were considered to be error-free plans. Therefore, the error detection rate of original plans represented the false error detection rate of the evaluated DVH action levels. The error detection rate of error-introduced plans represented the detectability of evaluated DVH-based action levels in identifying systematic MLC errors.

## Results

### QA Results and Process-Based DVH Action Levels

COMPASS measurement-based QA was implemented for all enrolled RapidArc plans, and QA results were acceptable (see **Table 1**). The average GPRs (3%/2mm, 10% dose threshold) were 99.23% ± 0.58%, and the mean GI (3%/2mm, 50% dose threshold) were 0.38 ± 0.04. Meanwhile, the linear accelerator was calibrated in every routine QA procedure to keep its output fluctuation within ±1%, and verification of the standard H&N RapidArc plan generated an average GI 0.44 ± 0.06, indicating a stable RapidArc delivery by linac during data collection.

**Table 1:** Summary of the descriptive statistics for QA results.

Group	Item	N	Mean ± SD
All enrolled plans	GPRs (3%/2mm, 10%)	148	99.23% ± 0.58%
	GI (3%/2mm, 50%)		0.38 ± 0.04
Standard H&N plan	GI (3%/2mm, 50%)	29	0.44 ± 0.06

**Figure 3:**
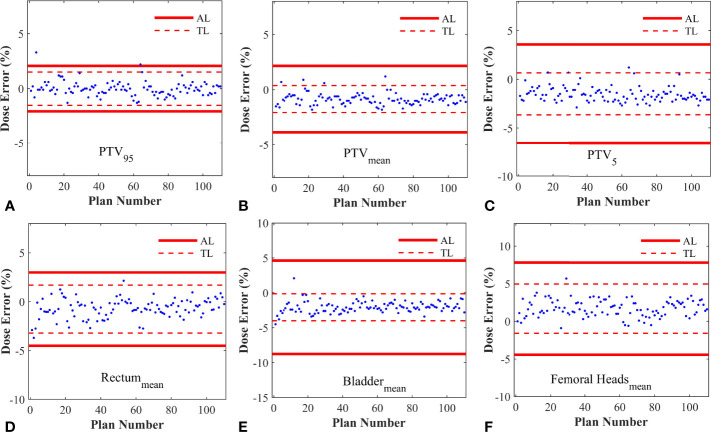
The control charts obtained from different DVH metrics. **(A)** The control chart of PTV_95_; **(B)** The control chart of PTV_mean_; **(C)** The control chart of PTV_5_; **(D)** The control chart of Rectum_mean_; **(E)** The control chart of Bladder_mean_; **(F)** The control chart of Femoral Heads_mean_. AL, Action limits; TL, Tolerance limits.

As described in **Table 2**, the %DE of DVH metrics in enrolled plans, which were selected to calculate process-based action levels, were within 3%. The Kolmogorov-Smirnov (K-S) test indicated that the %DE in all DVH metrics was in non-normal distribution except Bladder_mean_ and FH_mean_. Based on the QA results of 110 RapidArc plans, the DVH action levels were acquired, including action limits and tolerance limits. A summary of DVH-based action levels (AL and TL) in different DVH metrics was presented in **Table 2** and **Figure 3**.

**Table 2:** A list of DVH action levels obtained by statistical process control for each structure in RapidArc cervical cancer plans.

Structure	Metric	Mean ± SD (%)	K-S TEST	Action limits (%) [lower, upper]	Tolerance limits (%) [lower, upper]
PTV	D_5_	-1.53 ± 0.77	***p*=0.002**	[-6.56, 3.57]	[-3.65, 0.66]
	D_95_	-0.01 ± 0.67	***p*<0.001**	[-2.08, 2.07]	[-1.53, 1.51]
	D_mean_	-0.84 ± 0.53	***p*=0.006**	[-3.85, 2.16]	[-2.06, 0.38]
Bladder	D_mean_	-2.04 ± 0.83	***p*=0.011**	[-8.76, 4.63]	[-4.00, -0.14]
Rectum	D_mean_	1.73 ± 1.13	*p*=0.200	[-4.44, 7.82]	[-1.59, 4.97]
Femoral Head	D_mean_	-0.69 ± 0.99	*p*=0.056	[-4.51, 3.00]	[-3.21, 1.71]

The bold values indicate that the corresponding data are in non-normal distribution (p < 0.05).

### MLC Error Sensitivity With Different Action Levels

**Figure 4:**
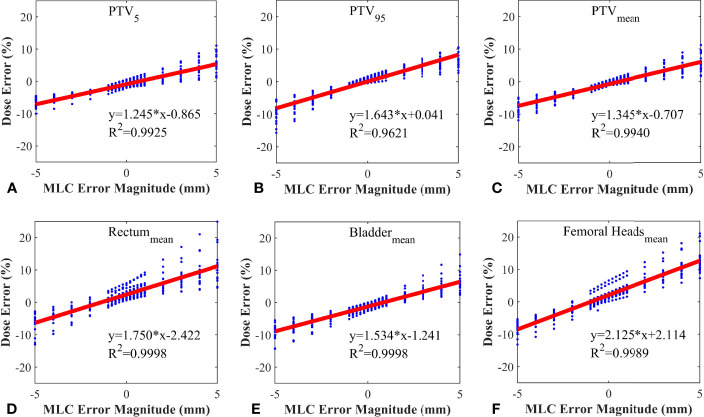
Correlation between dose error and systematic MLC error in different DVH metrics. **(A)** PTV_5_; **(B)** PTV_95_; **(C)** PTV_mean_; **(D)** Rectum_mean_; **(E)** Bladder_mean_; **(F)** Femoral Heads_mean_.

20 RapidArc plans were selected from the rest of plans, and systematic MLC errors of ±0.2mm, ± 0.4mm, ± 0.6mm, ± 0.8mm, ± 1mm, ± 2mm, ± 3mm, ± 4mm, ± 5mm were introduced in these plans. The %DE of DVH metrics were extracted from all the modified plans and original plans. Linear regression analysis was performed by having %DE of DVH metrics as function of the MLC error magnitude. The slope, y-intercept, 95% confidence interval and R^2^ were presented in **Table 3**. As shown in **Table 3** and **Figure 4**, the %DE of DVH metrics was found to have a linear dependence on the magnitude of systematic MLC errors when R^2^ was greater than 0.9. In addition, from the slope, FH_mean_ was found to be the most sensitive one to the MLC errors in all DVH metrics, and we also observed that PTV_95_ tended to be more sensitive to systematic MLC errors than other PTV DVH metrics.

**Table 3:** A list of the values obtained by linear regression for each structure.

Structure	Metric	Slope(%/mm)	y-intercept(%)	Lower 95% CI(%/mm)	Upper 95% CI(%/mm)	R^2^
PTV	D_5_	1.245	-0.865	1.191	1.299	0.9929
	D_95_	1.643	0.041	1.476	1.810	0.9621
	D_mean_	1.345	-0.707	1.291	1.398	0.9940
Bladder	D_mean_	1.534	-1.241	1.522	1.545	0.9998
Rectum	D_mean_	1.750	2.422	1.736	1.763	0.9998
Femoral head	D_mean_	2.125	2.114	2.098	2.161	0.9989

**Figure 5:**
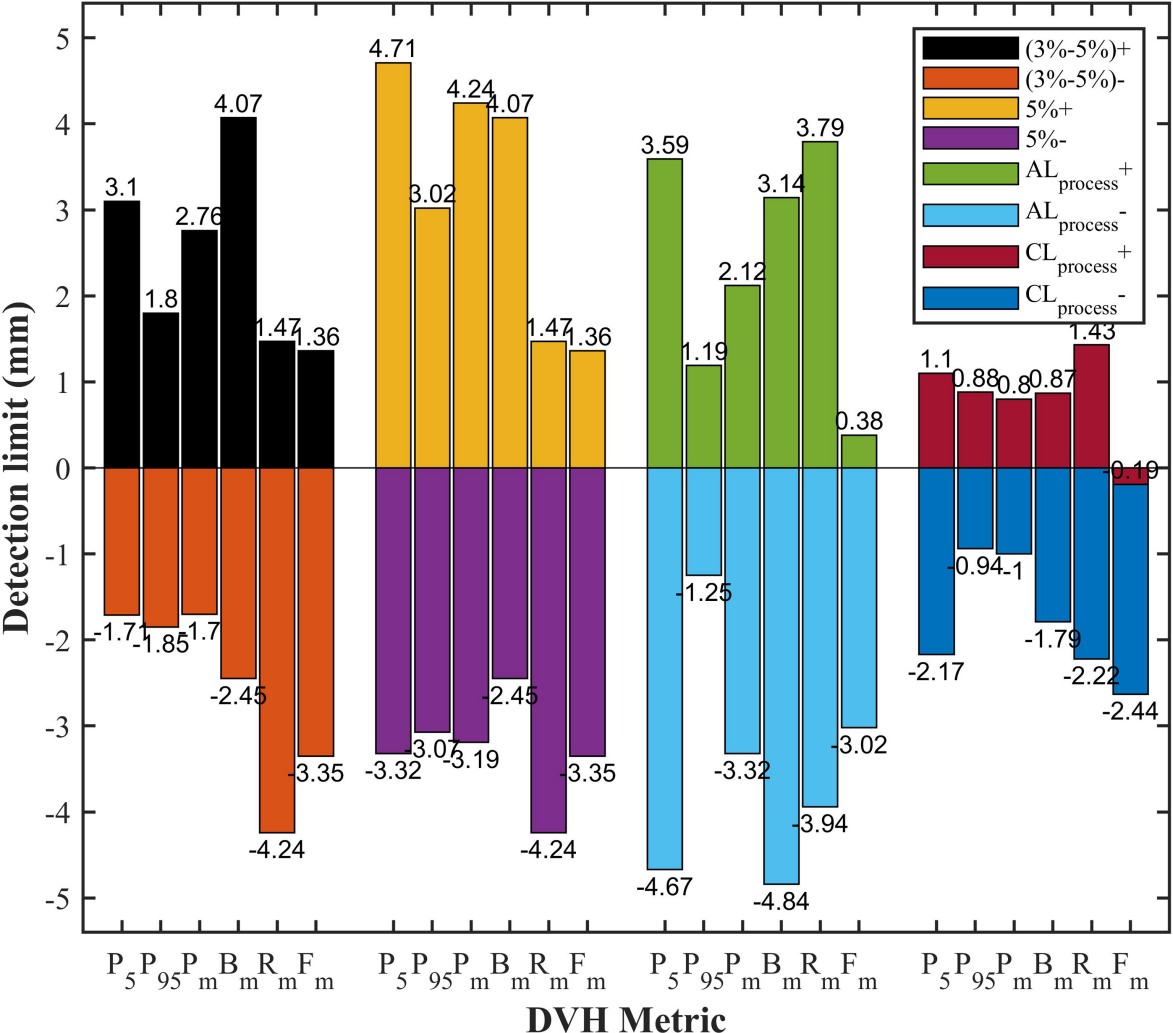
The comparison of detection limits in different DVH-based action levels. P_5_, PTV_5_; P_95_, PTV_95_; P_m_, PTV_mean_; B_m_, Bladder_mean_; R_m_, Rectum_mean_; F_m_, Femoral-heads_mean_; (3%-5%)+, 3% DVH action levels for target volume and 5% DVH action levels for OARs in positive direction; (3%-5%)-, 3% DVH action levels for target volume and 5% DVH action levels for OARs in negative direction; 5%+, 5% DVH action levels for target volume in positive direction; 5%-, 5% DVH action levels for target volume in negative direction; AL_process_+, process-based action limits in positive direction; AL_process_-, process-based action limits in negative direction; CL_process_+, process-based tolerance limits in positive direction; CL_process_-, process-based tolerance limits in negative direction.

All DVH-based action levels were input into linear regression model to calculate the theoretical detection limits. The detection limits represented the detectability of DVH-based action levels in catching system MLC errors. The smaller the detection limit, the better the detectability. As shown in **Figure 5**, the detection limits of tolerance limits were proven to be better than other DVH-based action levels. Meanwhile, PTV_95_ and PTV_mean_ had a superior detectability compared with other DVH metrics. As described in **Table 2** and **Figure 5**, the TL of PTV_95_ ([-1.54%, 1.51%]) could catch the systematic MLC errors between -0.94 mm and 0.88 mm, and the TL of PTV_mean_ ([-2.06%, 0.38%]) could also catch the systematic MLC errors between -1.00 mm and 0.80 mm. They were able to detect the systematic MLC errors greater than 1mm. It was worth noting that PTV_95_ action limits also showed a comparable detectability.

### Processed-Based DVH Action Levels Validation in Test Plans

**Figure 6:**
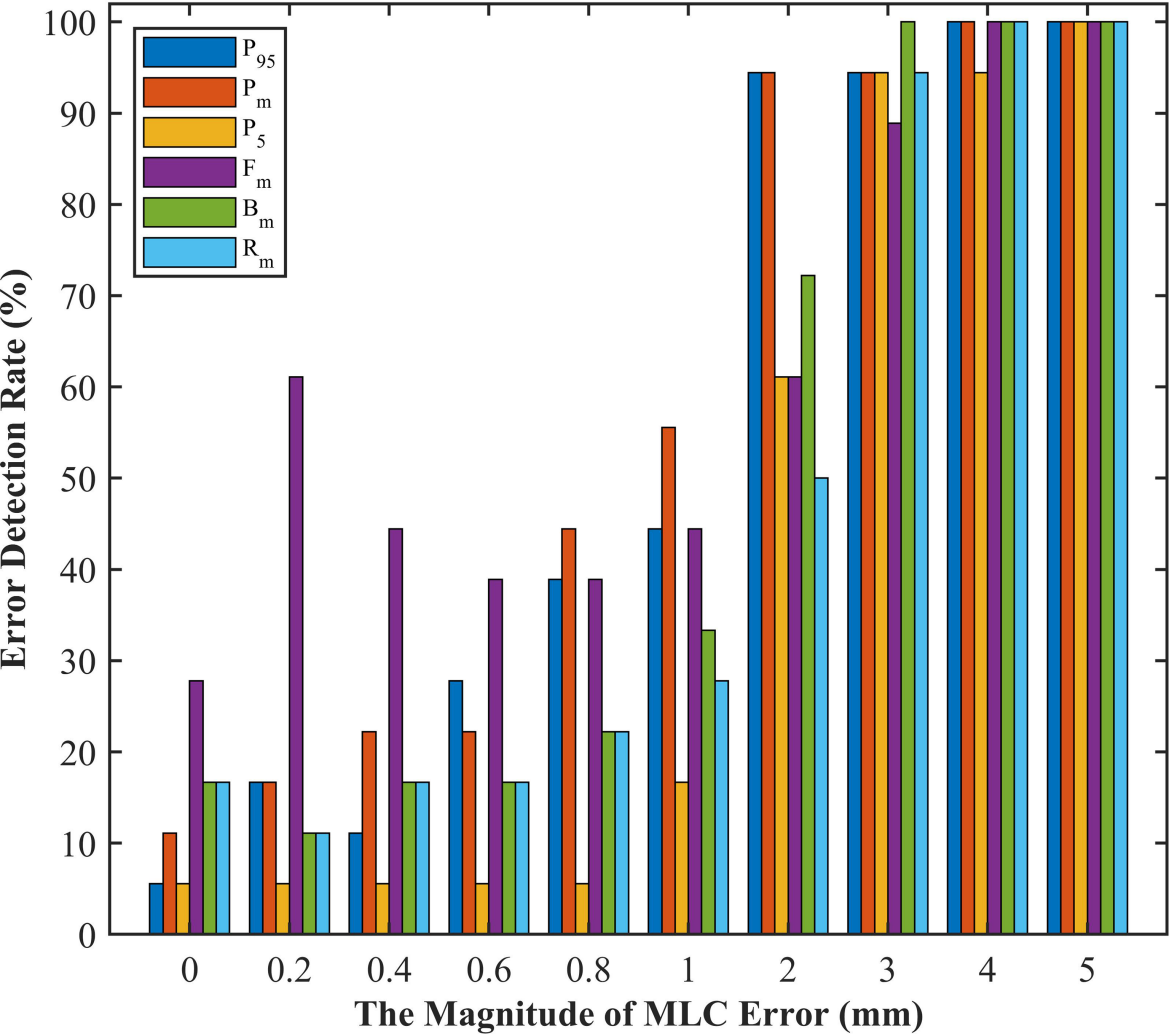
The error detection rate of test plans in different magnitude of systematic MLC errors. P_5_, PTV_5_; P_95_, PTV_95_; P_m_, PTV_mean_; B_m_, Bladder_mean_; R_m_, Rectum_mean_; F_m_, Femoral-heads_mean_.

Given that DVH-based tolerance limits had superior detection limits in identifying systematic MLC errors by linear regression model, we applied independent test plans to investigate whether the tolerance limits in different DVH metrics would effectively detect abnormal MLC delivery. Systematic MLC errors ranging from 0.2mm to 5mm were introduced in 18 RapidArc plans, and then 180 RapidArc plans (18 original plans plus 162 error-introduced plans) were generated to be evaluated by tolerance limits of different DVH metrics. The results were shown in **Figure 6** and **Table 4**. For original plans (0 mm MLC error), FH_mean_ showed the maximum error detection rate (27.8%), while PTV_95_ and PTV_5_ showed the minimum error detection rate (5.56%). For the error-introduced plans with systematic MLC errors less than 1mm, the tolerance limits of all DVH metrics were not able to identify systematic MLC errors effectively. FH_mean_ showed the maximum error detection rate of 45.83%, closely followed by PTV_mean_ (26.39%). For the error-introduced plans that systematic MLC errors were more than 1mm, PTV_mean_ had the maximum error detection rate of 88.89%. Of course, the error detection rate up to 94% demonstrated that the tolerance limits in all DVH metric were accurate to identify the error-introduced plans with systematic MLC errors more than 2mm.

**Table 4:** Error detection rate based on tolerance limits of different DVH metrics in different magnitude of systematic MLC errors.

Error magnitudes (mm)	Error detection rate (%)					
	P_95_	P_m_	P_5_	B_m_	R_m_	F_m_
0	5.56%	11.11%	5.56%	16.67%	16.67%	**27.78%**
0.2~0.8	23.61%	26.39%	5.56%	16.67%	16.67%	**45.83%**
1~5	86.67%	**88.89%**	73.33%	81.11%	74.44%	78.89%

P_5_, PTV_5_; P_95_, PTV_95_; P_m_, PTV_mean_; B_m_, Bladder_mean_; R_m_, Rectum_mean_; F_m_, Femoral-heads_mean_. The bold values indicate the best error detection rate in different magnitude of systematic MLC errors.

## Discussion

In this study, a comprehensive and systematic evaluation on DVH-based action levels was performed to cervical cancer RapidArc plans. The results demonstrated that process-based DVH action levels, especially tolerance limits, were more powerful than commonly-used DVH action levels in detecting systematic MLC errors in the patient-specific QA. With a long process, patient-specific QA includes random variations and systematic variations. Random variations refer to unavoidable random fluctuations in the process, and they should be monitored to confirm whether the process is under control (25). Arising from unpredictable, non-random events beyond the expected variability, systematic variations can be avoided (26). As an application of statistical techniques, SPC is able to distinguish random variations from systematic variations by specific control limits (also known as tolerance limits). If the data fall outside the control limits, it indicates that systematic errors may have been introduced in the process. That’s why tolerance limits have a greater ability to detect systematic MLC errors in patient-specific QA.

Actually, SPC has been proven to be an effective method to monitor and improve QA process using control charts for a long time (20, 21, 23, 26). A control chart typically consists of an upper control limit, a lower control limit and data points. Control limits are key parameters in control charts. As such, setting appropriate control limits is a critical step. Fuangrod T et al. (19) suggested that control limits should exclude clinically acceptable variability and focus on the detection of gross errors based on “good historical data”. Once the initial data contain a great deal of systematic variations, the control limits may be skewed and their ability in detecting systematic errors may be affected. For this reason, rigorous criteria should be set for selecting and collecting data. The QA results of enrolled RapidArc plans (**Table 1**) indicated that the average values of GPRs and GI met the criteria for the standard set. It is an effective way to eliminate systematic variations of initial data as much as possible. The initial data could also be affected by other factors, such as daily machine output fluctuations, setup variations and detector response variations. In order to minimize the variability introduced by these factors, routine QA procedure and a standard H&N RapidArc plan delivery was performed in every QA process. The QA results demonstrated that RapidArc plan’s delivery, QA measurements and equipment all remained in a stable state during data collection.

In addition, data characterization is a very important step in SPC charts. As shown in **Table 4**, the error detection rate of FH_mean_ in error-free plans reached as high as 27.78%. One reason was that the rejected plans did contain systematic errors. As shown in **Figure 5**, the detection limits in the same DVH metric were extremely asymmetrical in two directions and shifted from zero. M. steers et al. (17) concluded that the shifts in different direction may indicate different systematic errors caused by algorithm model, machine or other devices. The results of our study were also inevitably influenced by the deviation between AAA and CCC algorithm, the resolution limit of detector array, setup variations and other factors. That was why AAPM TG-218 highlighted that the systematic errors should be eliminated to the degree possible during the QA process. The other reason was that the DVH metrics were not all normally distributed (**Table 2**). Xiao et al. (18) have demonstrated that using normal-based control charts may result in incorrect decisions when the initial data is non-normally distributed. They have concluded that applying a normal-based control chart to a non-normal distribution process could increase the type risk and false alarm rate. The solution was to either transform the non-normal data into normal data, or to use a non-normal-based method to obtain the control chart.

After filtering the data and characterizing the data distribution, the control limits (or tolerance limits) were established by repeating the “Identify-Eliminated-Recalculate” procedure several times (18). The QA results within tolerance limits implied that the process was only affected by random errors and considered to be under control. As long as the process is under control, action limits are also calculated from QA measurement results over a time period. As shown in **Table 2**, action limits in each DVH metrics were more lenient than tolerance limits. It is because that action limits are set as a minimum level of process performance, and defined as the boundaries outside which a process could cause a negative clinical impact for the patient (13). Therefore, the last step in patient-specific QA process is to compare tolerance limits with action limits, and to ensure that the tolerance limits are within the action limits. Furthermore, it was worth noting that differences in the tolerance limits and action limits were observed between different structures and DVH metrics. It is clear that universally action levels are not adequate for patient-specific QA, especially when individualized evaluation is drawing more and more attention in QA process. Above all, there are variations in QA process due to different factors, but SPC provides a valuable and specific method to identify these variations, ensuring the precision of treatment delivery and safety of patients in radiotherapy.

Gantry, collimator, dose rate and MLC are not only the crucial modulated parameters in RapidArc plans, but also the root cause of variations in QA process. T. Betzel et al. (27) have demonstrated that RapidArc plans are more susceptible to MLC variations rather than incorrect gantry or collimator angle, and dose rate variations. Moreover, MLC plays a decisive role in the beam modulation, therefore, high-quality radiation therapy requires optimal MLC (28, 29). Small errors in MLC positioning may lead to an adverse consequence to dose distribution, so the accuracy of MLC leaf positions should be monitored carefully. However, a considerable number of studies have reported that the dosimetric impact caused by individual MLC positioning offsets or random MLC errors is relatively insignificant. Wang et al. (24) have also reported that MLC positional errors have a distinguishable dosimetric effect relative to other linac errors. It was not hard to understand why our study focused on the effect of the systematic MLC positional errors in DVH metrics. As shown in **Table 3**, the dose sensitivity of systematic MLC errors was 1.643%/mm, 1.345%/mm for PTV_95_ and PTV_mean_, respectively. These results can be compared with that in previous studies (29, 30). The FH_mean_ was found to have the highest dose sensitivity of 2.15%/mm. It may be attributed to the fact that femoral heads were located in low-dose regions, thus the relative difference was magnified. More importantly, we observed a strong positive linear correlation between DVH metrics and systematic MLC errors when R^2^ was greater than 0.9. It was consistent with values previously reported in other studies (30–32). The strong relationship between DVH metrics and systematic MLC errors implied that reasonable DVH-based action levels could identify and eliminate the systematic MLC errors to the degree possible. However, the comparison of detection limits indicated that commonly used DVH-based action levels were insensitivity enough to detect systematic MLC errors with clinically significant DVH differences. A. Sdrolia et al. (4) have reported that action levels should not be blindly copied, and they must be locally determined based on tumor response and normal tissue complication. So, how to select reasonable DVH-based action levels was one of the issues that should be solved in our study.

It has been suggested in a number of literatures (10, 32, 33) that the deviations in DVH metrics for target volume should be kept within ±2%. The tolerance limits of PTV_95_ and PTV_mean_ in our study were consistent with this requirement. As described in **Figure 6** and **Table 4**, the comparison of detection limits and error detection rate demonstrated that systematic MLC errors up to 1mm could be effectively caught by tolerance limits. In numerous publications (6, 13, 29, 34), it has been found that the systematic MLC errors up to 1mm can produce a clinically relevant influence on the dose distribution, but leaf position errors less than 2mm is unable to be detected by traditional gamma analysis with commonly used criterion of 3%/3mm. It has also been recommended in AAPM TG-142 report that the leaf position accuracy should be within ±1mm (35). Therefore, process-based DVH action levels, especially tolerance limits, can not only detect systematic MLC errors with clinical significance, but also have a superior detectability than commonly used action levels. Notably, the highest error detection rate in detecting plans with systematic MLC errors less than 1mm was only 45.83% (FH_mean_). However, Rangel et al. (36) have suggested that systematic MLC errors need to be limited to 0.3mm. Oliver et al. (32) have suggested that the systematic MLC errors should be within 0.63mm to keep the PTV95 within 2%. As for the low efficiency in catching minor systematic MLC errors, one factor was that the detectability was limited to the low-spatial resolution array detectors. Woon et al. (37) have also found that a 0.75mm systematic MLC error is undetected due to the poor resolution of array detectors. Another factor was that the sensitivity to MLC errors could vary according the treatment sites, delivery techniques or QA measurement instruments. In addition, the introduced MLC errors in our study were systematic MLC shift errors and tended to have a smaller effect on the DVH metrics than systematic open/closed MLC errors (38). Given the above, the insensitivity of tolerance limits to detect systematic MLC errors below 1mm was reasonable, and process-based DVH action levels may offer reasonable action levels to identify and catch systematic MLC errors with clinical significance.

Once the method for obtaining DVH-based action levels was determined, the next challenge was to select appropriate DVH metrics from numerous evaluation structures and make a final QA determination. It is widely known that MLC positional errors can lead to clinically significant dose deviation in the PTV and adjacent OARs. Many studies (1, 4, 32, 39) have demonstrated that DVH metrics of PTV are the primary concern with the quality and deliverability of VMAT plans. The dose distribution in the PTV is more sensitive to MLC positional errors because that PTV is mostly deep-seated and close to isocenter (40), and MLC apertures varies dynamically along with the shape of PTV. As described in **Table 4**, PTV_95_ showed a higher error detection rate in plans with systematic MLC errors greater than 1mm, but a lower error detection rate in original (error-free) plans. This implied that PTV_95_ may be a representative DVH metric in detecting systematic MLC errors in this study. The change of DVH metrics in OARs depended on the location of OARs relative to the target volume and the size of OARs. The dose sensitivity of OARs in this study was in agreement with the study of Nithiyanantham et al. (29) that the OARs partially located within target volume or having a smaller volume showed an obvious dose difference in DVH metrics with MLC positional errors. However, our previous study has found that the OARs with smaller volume or far away from the isocenter could generate false positive results. Therefore, after completing the evaluation of target volume, it is necessary to carry out an objective analysis to the dose difference of OARs in DVH metrics.

Of course, there are some limitations in this study. First of all, the MLC positional errors included in this study were over-simplified. MLC positional errors were correlated with the MLC speed, gantry sag and other factors. In the future, more machine errors need to be simulated and introduced to investigate the dose sensitivity to these errors. Secondly, the data of DVH metrics were not all normally distributed. The non-normal distribution may result in incorrect decisions for DVH-based action levels. Further research is needed to address issue of non-normal distribution so as to obtain more reliable action levels by SPC. Finally, our findings may be confined to our devices, treatment sites and sample size. However, process-based DVH action levels could be available to other delivery techniques, treatment sites or QA tools. A large sample size is required and further investigation need to be implemented for other dosimetric system or treatment sites.

## Conclusions

In conclusion, for the cervical cancer RapidArc plans, through tolerance limits based on statistical process control, reasonable DVH-based action levels can be acquired to identify and catch systematic MLC errors more than 1mm in 3D dose verification. During the evaluation of DVH metrics, a comprehensive analysis focusing on target volume should be implemented on structure by structure in order to ensure the quality and deliverability of radiotherapy plans. Although the OARs in low-dose regions showed a relatively stronger dose sensitivity to systematic MLC errors, their process-based DVH action levels may be accompanied with higher false error detection rate.

## Data Availability Statement

The original contributions presented in the study are included in the article/supplementary material. Further inquiries can be directed to the corresponding author.

## Author Contributions

HZ and WL have contributed equally to this work. XY, HZ and WL: drafting of work, analysis and interpretation of trials and literature, drafting of manuscript, and manuscript review. HZ, XY collected the data, reviewed the literature, and wrote the paper. HC and YL prepared the figure and contributed in the revision of the literature. All authors contributed to the article and approved the submitted version.

## Funding

This work was sponsored by Natural Science Foundation of Chongqing, China (cstc2021jcyj-msxmX0138).

## Conflict of Interest

The authors declare that the research was conducted in the absence of any commercial or financial relationships that could be construed as a potential conflict of interest.

## Publisher’s Note

All claims expressed in this article are solely those of the authors and do not necessarily represent those of their affiliated organizations, or those of the publisher, the editors and the reviewers. Any product that may be evaluated in this article, or claim that may be made by its manufacturer, is not guaranteed or endorsed by the publisher.
